# Ultrasonographic Characteristics in the Fingers and Other Superficial Glomus Tumours

**DOI:** 10.1155/2023/7126799

**Published:** 2023-07-26

**Authors:** Noboru Takanashi, Satomi Asai, Yoko Ogase, Akiko Fujii, Haruyo Atsumi, Mika Doi, Nobue Kumaki, Tomotaka Mabuchi, Hayato Miyachi

**Affiliations:** ^1^Department of Clinical Laboratory Technology, Tokai University Hospital, 143 Shimokasuya, Isehara, Kanagawa 259-1196, Japan; ^2^Department of Laboratory Medicine, Tokai University School of Medicine, 143 Shimokasuya, Isehara, Kanagawa 259-1196, Japan; ^3^Department of Pathology, Tokai University School of Medicine, 143 Shimokasuya, Isehara, Kanagawa 259-1196, Japan; ^4^Department of Dermatology, Tokai University School of Medicine, 143 Shimokasuya, Isehara, Kanagawa 259-1196, Japan

## Abstract

Glomus tumours are painful superficial tumours, and ultrasonography is an extremely useful and noninvasive diagnostic technique for superficial organs. In this study, we retrospectively examined glomus tumours using ultrasonography. Among 18 patients histopathologically diagnosed with glomus tumours via ultrasonography, we observed five different development sites: subungual areas or those surrounding the nail bed (12), other areas on the finger surface (3), abdominal wall (1), upper arm (1), and forearm (1). The ultrasonographic images revealed significant differences in tumour size, indicating that tumours on other body surfaces tended to be smaller than those on patients' fingers (*p* < 0.01). The depth/width ratios of tumours on the other body surfaces were significantly higher than those on the fingers (*p* < 0.05). The tumours showed a regular shape (72.2%) and clear border (100%). Furthermore, most tumours were low-echo tumours with a diameter of up to 15 mm, clear margins, and no lateral shadows. Abundant blood flow and vessels in and out of the tumours were also observed. In conclusion, our study describes the ultrasonographic characteristics of glomus tumours and reveals that they cannot be ruled out when diagnosing small painful subcutaneous tumours.

## 1. Introduction

Glomus tumours were first described by Masson, who observed a specialised form of arteriovenous anastomosis in the peripheral areas of the skin and described it as a glomus neuromyoartrial formation, in 1924 [1]. As the histological images were similar to those of tumours, these nodules were originally described as glomus tumours or arterial angioneuromyoma [1]. Glomus tumours are relatively rare, benign, vascular tumours that can either be classified as solitary or multiple tumours (with the former accounting for the majority of cases) [2–4]. Solitary glomus tumours frequently develop in subungual areas and are accompanied by spontaneous pain, which tends to intensify with compression, cold, and other factors [5, 6]. Indeed, glomus tumours should be primarily suspected when a painful subungual tumour is observed. However, tumours that grow on body surfaces other than subungual areas must first be differentiated from other types of superficial, painful tumours rather than directly classified as glomus tumours [7–10]. Such diagnoses are typically performed through histopathological examination by biopsy; therefore, the ultrasonographic characteristics of such tumours remain unclear [6]. Nevertheless, ultrasonography is an extremely useful and noninvasive diagnostic aid for superficial organs such as the lymph nodes, breasts, and lips [11–14]. Hence, in this study, we retrospectively examine the ultrasonographic characteristics of glomus tumours, including those found on nonsubungual body surfaces.

## 2. Materials and Methods

### 2.1. Subjects

All patients provided verbal and written informed consent to participate in this study. Furthermore, the study was approved by the review board of Tokai University (18R192). The subjects included 18 patients (four men and fourteen women; mean age, 54 (range, 23–82) years) who were histopathologically diagnosed with glomus tumours following ultrasonography on painful superficial tumours in Tokai University Hospital from April 2012 to December 2018. This study only involved patients with solitary tumours. The following ultrasonography devices were used on the subjects: TUS-A300, TUS-A500, and SSA-790A (Canon Medical Systems Corporation). The following probes were employed, all with a frequency range of 5–18 MHz: PLT-1204BX, PLT-1204BT, and PLT-1204BX.

### 2.2. Study Procedure

The following tumour-related features were retrospectively examined with reference to ultrasonographic images: sites of development, maximum diameter, depth/width ratio, shape, border, lateral shadow, internal echo, posterior echo, blood flow, and high echo surrounding the tumour. Tumour shapes were either round, squamous, or elliptical, whereas tumours with corners and constrictions were considered to be irregular. Tumours with a depth/width ratio of less than 0.5 were deemed to be squamous in shape. A clear border refers to the boundary between the tumour and the surrounding tissue; if the tumour and surrounding tissue could not be distinguished, the border was considered unclear, with a sectional border being partially unclear. Lateral shadows could occur on both or one side of the tumour. Blood flow was evaluated using colour Doppler or power Doppler methods. Tumours were categorised according to the proportion of a single image attributed to blood flow, i.e., a high blood-flow pattern indicated that blood flow was observed throughout the tumour; a low blood-flow pattern indicated that blood flow was observed in up to 50% of the total tumour area; and a moderate blood-flow pattern indicated blood flow between these two extremes. Moreover, for five patients who were subjected to blood-flow velocity measurement using the pulse Doppler method, we examined the presence of blood vessels flowing in and out of the tumour and measured the maximum blood flow velocity. Ultrasonographic images were evaluated by a registered medical sonographer for superficial regions certified by the Japan Society of Ultrasonics in Medicine with >10 years of experience. Welch's *t*-test was used to test for significance, and the statistical significance was set to *p* < 0.05 or *p* < 0.01.

## 3. Results

### 3.1. Developmental Sites

Twelve patients had glomus tumours in subungual regions or areas surrounding the nail bed (67%), which accounted for the most common developmental site. The remaining six glomus tumours occurred on the finger surface (specifically on the thumb) in three patients (17%), the abdominal wall in one patient (6%), the upper arm in one patient (6%), and the forearm in one patient (6%). Thus, glomus tumours developed on the fingers in 15 out of 18 patients (83%).

### 3.2. Tumour Size and Depth/Width Ratio

The mean maximum diameter was 6.8 mm (3–15 mm). Tumour diameters of ≥10 mm, 5–10 mm, and <5 mm were observed in four patients (22.2%), seven patients (38.9%), and seven patients (38.9%), respectively. Tumours <10 mm accounted for 77.8% of all tumours. No tumours ≥10 mm were observed on body parts other than the fingers. According to ultrasonographic analysis, the maximum tumour diameter in the 15 patients with tumours of the finger was 3–15 mm (mean, 7.3 mm), whereas that in the other three patients was 4–5 mm (mean, 4.3 mm). Welch's *t*-test showed a significant difference in the maximum diameter between tumours on the fingers and those on other parts of the body (*p* < 0.01) (Table 1).

The mean depth/width ratio for all patients was 0.58 (0.29–1.0). For the fifteen patients with tumours of the finger, the mean depth/width ratio was 0.53. For the three patients with tumours on other body surfaces, the mean depth/width ratio was 0.83 (Table 1). The depth/width ratios of tumours on the other body surfaces were significantly higher than those on the fingers (*p* < 0.05). We then compared the six tumours on patients' fingers that were ≦5 mm with the three tumours on other body surfaces and observed that the mean maximum diameters were 0.65 and 0.83, respectively, However, no significant difference was observed between the two groups (*p* = 0.13) (Table 2).

## 4. Shape

**Figure 1:**
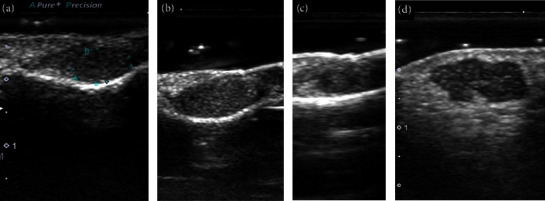
Representative ultrasound images of tumour shapes: (a) regular (ellipse), (b) regular (squamous), (c) regular (round), and (d) irregular.

The tumour shape was regular in 13 patients (72%) and irregular in five patients (28%). Among the regular tumour shapes, five were elliptical, six were squamous, and two were round (Figure 1). Clear and partially unclear borders were observed in seventeen patients (94%) and one patient (6%), respectively. Most of the finger masses were elliptical or squamous: elliptical in seven patients, round in one patient, squamous in four patients, and irregular in three patients. Other glomus tumours on the body surface were small spherical or irregularly shaped masses with a higher depth/width ratio.

### 4.1. Lateral Shadow

Lateral shadow was nondeterminable in one patient because of bone deformity. Thus, excluding this patient, tumours accompanied by a weak lateral shadow were observed in two patients (12%), whereas fifteen patients had a tumour with no lateral shadow (88%), representing the majority of tumours.

### 4.2. Internal and Posterior Echo

**Figure 2:**
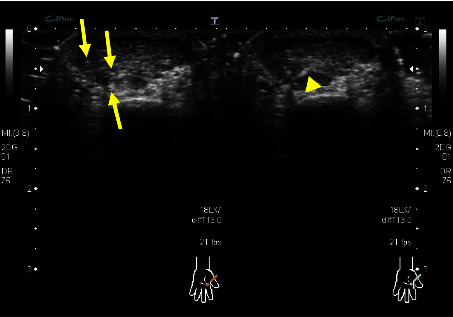
Example of fine echo spots (arrows) and hyperechoic strands (arrowheads) observed inside a tumour (patient 2).

The internal echo of solid hypoechoic tumour was even in fourteen patients (78%) and uneven in four patients (22%). Fine echogenic spots were observed internally in three patients (Figure 2). No tumours were accompanied by cystic portions internally. Posterior echo was enhanced in fourteen patients (78%) and unchanged in four patients (22%). None of the tumours were attenuated. Moreover, the degree of enhancement was small in seven of the fourteen patients with enhanced posterior echo.

### 4.3. Blood-Flow Evaluation

**Figure 3:**
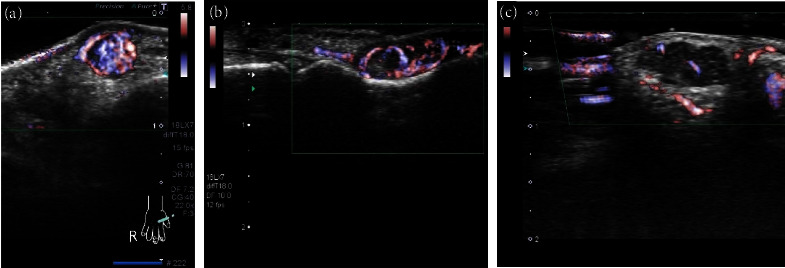
Ultrasound images of blood-flow patterns: (a) high blood flow, (b) moderate blood flow, and (c) low blood flow.

**Figure 4:**
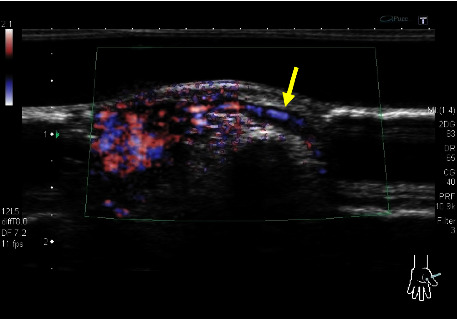
Ultrasound image of vessels flowing in and out of a tumour (yellow arrow).

The blood-flow patterns observed by power or colour Doppler ultrasound were high in six patients (33%), moderate in eleven patients (61%), and low in one patient (6%) (Figure 3). In addition, vessels flowing in and out of the tumour were observed in fourteen patients (78%) (Figure 4). The maximum blood flow velocity in five patients whose blood inflow and outflow was measured via the pulse Doppler method was 5.0–13.8 cm/s (mean, 7.7 cm/s) (Table 3).

### 4.4. High Echo Surrounding the Tumour

**Figure 5:**
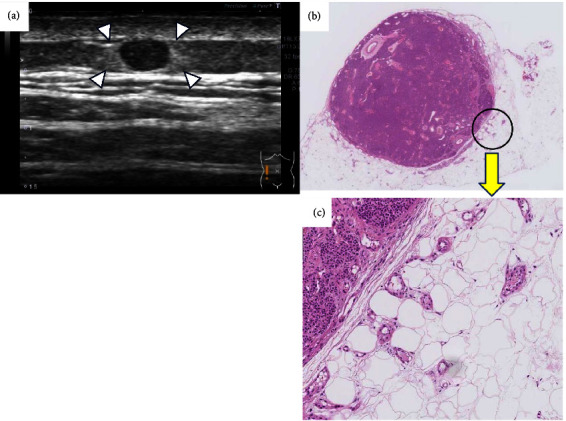
High echo surrounding a glomus tumour that developed on the abdominal wall: (a) ultrasound image of the tumour. The image showed high echo with indistinct boundaries around the tumour in the subcutaneous fat layer (arrowheads), (b) histopathological tissue (magnified image) revealed a nodule with clear margins, which were observed within the adipose tissue, and densely packed cubic tumour cells enlarged to an oval shape proliferated in a cobblestone-like pattern, and (c) enlarged image of the tumour (areas surrounding the tumour) showed that small diffuse vessels were observed within the adipose tissue of areas surrounding the nodule.

High echo surrounding the tumour was observed in two patients (11%). This high echo was observed in the subcutaneous fat of tumours that grew on body surfaces other than the fingers. For example, in the glomus tumour that developed on the abdominal wall, high echo with unclear margins around the tumour was observed in the subcutaneous fat layer (Figure 5). Histopathological examination revealed a nodule with clear margins within the adipose tissue, and densely packed cubic tumour cells enlarged to an oval shape proliferated in a cobblestone-like pattern (top left). In enlarged magnification, small diffuse vessels were observed within the adipose tissue of areas surrounding the nodule (lower left).

## 5. Discussion

In this study, we retrospectively analysed ultrasonographic images of glomus tumours located in subungual or other areas in 18 patients. The most frequent sites of development were the subungual regions and areas surrounding the nail bed. Tumours on the fingers, including the thumb, were noted in 83% of patients in this study. Glomus tumours are painful tumours that frequently grow subungually but can also grow on other areas of the body. A previous analysis of tumour development sites in 126 patients reported the following proportions: 66% on the hand, 11% on the forearm, 9% on the thigh, 5% on the upper arm, 3% on the lower leg, 2% on the foot, 2% on the head and neck, and 1% on the trunk [15]. These results suggest that glomus tumours do not only refer to painful solid tumours on the fingers. Our study confirmed these previous findings, that is, glomus tumours were observed in areas other than the fingers in three patients (i.e., abdominal wall, upper arm, and forearm). However, a differential diagnosis by ultrasonography for these three patients may not typically include glomus tumours. Thus, despite their low incidence, it is necessary to also consider glomus tumours in the differential diagnosis of painful subcutaneous tumours that grow in areas other than the fingers.

The ultrasonographic images revealed significant differences in the mean maximum diameter of tumours, indicating that tumours on other body surfaces of patients tended to be smaller than those on their fingers (*p* < 0.01). The ultrasonographic images revealed significant differences in tumour mean depth/width ratio, indicating that the tumours on patients' fingers tended to be smaller than those on other body surfaces (*p* < 0.05). These results suggest that finger glomus tumours tend to be elliptical or rather flat, whereas tumours on other body surface tend to have a high depth/width ratio.

**Figure 6:**
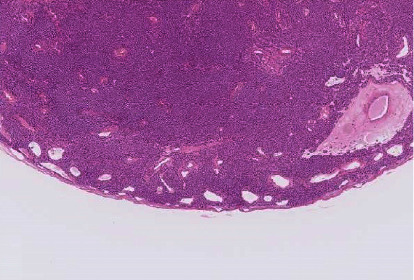
A thin fibrous capsule observed in a glomus tumour.

A lateral shadow was observed in only two patients (88%). A lateral shadow is an image artifact wherein the lateral posterior side of the tumour is missing; this occurs when the speed of sound inside the tumour is slower than that in the surrounding tissue. In this case, the sound wave which travels into the tumour is refracted and converges inward, whereas the rest of the sound wave is reflected upon impact at the critical angle in areas near the boundary of the spherical tumour, thereby leading to a decrease in the transmitted wave in these areas. Ultrasonic wave refraction is greater in cysts and round-shaped tumours and tends to cause lateral shadows. In tumours with irregular borders, a lateral shadow is unlikely because the ultrasonic waves are scattered. Although the tumours examined in this study were not fully compared with pathological tissue samples, fibrous capsules, including partial fibrous capsules, were observed in 15 of 16 patients (assessment could not be performed in two of the patients). Most of these fibrous capsules were thin (Figure 6); a relatively thick and even fibrous capsule was observed in one patient only, who had a small tumour with a maximum diameter of 3 mm. The two tumours with lateral shadows were covered by a fibrous capsule, which had diameters of 15 mm and 4 mm. The lack of lateral shadows was attributed to the small difference in the speed of sound between the tissue surrounding the tumour and that inside the tumour and the limited impact of the critical angle generally caused by a small tumour size.

Solid tumours were observed in fourteen patients (78%) with an even internal echo. Uneven internal echo was observed in four patients, of which three had a tumour with fine echo spots or hyperechoic strands internally. No tumours had an acoustic shadow, which may be because relatively thick vessel walls and collagen fibres were drawn in the form of high-echo intensity caused by posterior scattering. Enhanced posterior echo was observed in fourteen patients (78%); this was attributed to the increased permeability caused by a limited decrease in ultrasonic waves from a decrease in the difference in acoustic impedance, which reflects the proliferation of sheet- and cord-like tumour cells and mucus-like substrates.

Blood-flow patterns inside the tumours were evaluated using colour Doppler or power Doppler methods. A moderate blood-flow pattern, which indicated the presence of blood flow in at least 50% of the tumour, was observed in 17 patients (94%). Thus, abundant blood-flow signals were observed in all tumours. Fan et al. studied 62 patients and showed abundant blood-flow signals in 38.7% (24/62) of tumours, low blood-flow signals in 35.5% (22/62) of tumours, and no blood-flow signals in 19.4% (16/62) of tumours. The absence of blood-flow signals occurred in tumours with few blood vessels, i.e., glomangiomyoma or myxoid glomus tumours, wherein dilated vessels were accompanied by thrombus [16]. In our study, none of the 18 patients had glomangiomyomas. Moreover, blood-flow signals tend to be low in the glomus and myxoid tumour subtypes; thus, pain is a typical clinical symptom in these tumour types. However, histological changes may occur in glomus tumours with low blood-flow signals. Vessels flowing in and out of the tumour were observed in 14 patients (78%). Maximum velocities were measured in five patients by the pulse Doppler method, which revealed low velocities with pulsation waves of 5.0–13.8 cm/s (mean, 7.7 cm/s). A study by Wortsman et al. examined 13 patients with glomuvenous malformation, including multiple cases, using the Doppler method [17]. They observed low velocities of up to 15 cm/s in 85% of the 13 patients included in their study. Fan et al. reported maximum velocities of 4.9–25.6 cm/s for 46 patients [16]. However, previous studies did not provide mean or median values. In the five patients examined in this study, pulsation waves were noted in the tumours, with a maximum velocity of 15 cm/s considered as an indicator of glomus tumours.

High echo around the tumours were observed in only two patients, who had tumours on body surfaces other than the fingers, specifically, on the upper arm and abdominal wall. High echo around a breast cancer tumour is known as a halo; this high echo is observed in the lesion margin and indicates breast cancer cell infiltration into the adipose tissue in the breast cancer margin. The occurrence of a halo may be attributed to strong posterior scattering resulting from the difference in the speed of sound waves between cancer cells, which contain a large amount of water and adipocytes [18]. Using ultrasound images, Tohno and Bando observed weak echogenic areas in the breast cancer margin in the absence of cancer cell infiltration into the surrounding adipose tissue [19]. These researchers also observed high echo in oedema-like functional changes caused by lymphostasis, which they referred to as a pseudohalo. In the two patients in our study, marked small vessels were observed in one patient and connective tissue proliferation in the other. We assume that posterior scattering occurred due to the proliferation of small vessels and connective tissue within the adipose tissue, which in turn, resulted in high echo in areas surrounding the tumours.

Therefore, we used ultrasonography to show that solitary glomus tumours are low-echo tumours with a diameter of up to 15 mm, exhibiting clear margins and a lack of lateral shadow. Blood flow inside the tumours was abundant, with frequent vessels flowing in and out of the tumours. Thus, glomus tumours should be considered in the differential diagnosis of painful tumours ≤15 mm wherein blood-flow signals are observed.

## 6. Conclusion

This study constituted a retrospective analysis of ultrasonographic images containing solitary glomus tumours. The results revealed that most tumours were low-echo tumours with a diameter of up to 15 mm, clear margins, and no lateral shadows. Abundant blood flow and vessels flowing in and out of the tumours were also observed. Moreover, the size of glomus tumours in areas other than the fingers was 5 mm or less; however, their depth/width ratio was greater than that of tumours of the fingers. Our study describes the ultrasonography characteristics of glomus tumours and reveals the importance of considering glomus tumours in the differential diagnosis of small painful subcutaneous tumours.

## Figures and Tables

**Table 1:** Maximum tumour diameter and depth/width on ultrasonographic images.

	Maximum diameter (mm)			Depth/width		
	Minimum	Maximum	Mean	Minimum	Maximum	Mean
All patients (*n* = 18)	3	15	6.8	0.29	1.00	0.58
Fingers (*n* = 15)	3∗	15^*∗*^	7.3^*∗*^	0.29^*∗∗*^	0.80^*∗∗*^	0.53^*∗∗*^
Other body surfaces (*n* = 3)	4^*∗*^	5^*∗*^	4.3^*∗*^	0.75^*∗∗*^	1.00^*∗∗*^	0.83^*∗∗*^

^*∗*^ *p* < 0.01; ^*∗∗*^*p* < 0.05.

**Table 2:** Maximum diameter and depth/width of tumours ≤5 mm.

	Maximum diameter (mm)			Depth/width		
	Minimum	Maximum	Mean	Minimum	Maximum	Mean
Fingers (*n* = 6)	3	5	3.8	0.50∗	0.80^*∗*^	0.65^*∗*^
Other body surfaces (*n* = 3)	4	5	4.3	0.75^*∗*^	1.00^*∗*^	0.83^*∗*^

^*∗*^ *p* = 0.13.

**Table 3:** Results of pulse Doppler analysis performed on five patients.

Site	Maximum diameter (mm)	Depth/width	Blood-flow pattern	Image of vessels flowing in/out	Maximum velocity (cm/s)
Palmar side of the proximal phalanx of the left first digit	15	0.47	High	Yes	5.0
Nail root of the left third digit	4	0.50	High	No	5.7
Subungual area of the left first digit	11	0.45	High	Yes	13.8
Dorsal side of the right upper arm	6	0.83	Medium	Yes	5.0
Pad of the right first digit	7	0.29	Medium	Yes	9.0

## Data Availability

All data used to support the findings of this study are included within the article.
